# A Review of A Priori Regression Models for Warfarin Maintenance Dose Prediction

**DOI:** 10.1371/journal.pone.0114896

**Published:** 2014-12-12

**Authors:** Ben Francis, Steven Lane, Munir Pirmohamed, Andrea Jorgensen

**Affiliations:** 1 Department of Biostatistics, University of Liverpool, Liverpool, United Kingdom; 2 Department of Biostatistics, University of Liverpool, Liverpool, United Kingdom; 3 Department of Molecular and Clinical Pharmacology, University of Liverpool, Liverpool, United Kingdom; 4 Department of Biostatistics, University of Liverpool, Liverpool, United Kingdom; Michigan State University, United States of America

## Abstract

A number of a priori warfarin dosing algorithms, derived using linear regression methods, have been proposed. Although these dosing algorithms may have been validated using patients derived from the same centre, rarely have they been validated using a patient cohort recruited from another centre. In order to undertake external validation, two cohorts were utilised. One cohort formed by patients from a prospective trial and the second formed by patients in the control arm of the EU-PACT trial. Of these, 641 patients were identified as having attained stable dosing and formed the dataset used for validation. Predicted maintenance doses from six criterion fulfilling regression models were then compared to individual patient stable warfarin dose. Predictive ability was assessed with reference to several statistics including the R-square and mean absolute error. The six regression models explained different amounts of variability in the stable maintenance warfarin dose requirements of the patients in the two validation cohorts; adjusted R-squared values ranged from 24.2% to 68.6%. An overview of the summary statistics demonstrated that no one dosing algorithm could be considered optimal. The larger validation cohort from the prospective trial produced more consistent statistics across the six dosing algorithms. The study found that all the regression models performed worse in the validation cohort when compared to the derivation cohort. Further, there was little difference between regression models that contained pharmacogenetic coefficients and algorithms containing just non-pharmacogenetic coefficients. The inconsistency of results between the validation cohorts suggests that unaccounted population specific factors cause variability in dosing algorithm performance. Better methods for dosing that take into account inter- and intra-individual variability, at the initiation and maintenance phases of warfarin treatment, are needed.

## Introduction

Warfarin is the most commonly used anticoagulant in the UK, with an estimated 1% of the population currently undergoing warfarin therapy [1]. The aim of warfarin therapy is to bring the International Normalized Ratio (INR), a measure of the patients clotting capability, within therapeutic range, and to maintain it within that range. Although warfarin is an effective anticoagulant, determining the dose required, after loading and refinement phases of warfarin therapy, to achieve a stable therapeutic INR (the “stable maintenance dose”) is difficult due to the large inter-individual variability in maintenance dose requirements, and warfarin's narrow therapeutic index.

Therapeutic INR range is typically 2 to 3 for most patients on warfarin; outside this range adverse events are more likely to occur [2]. If the concentration of warfarin in the body is too low then the drug will not provide the desired therapeutic effects, leading to a risk of thrombosis. Conversely, if the amount of warfarin in the body is too high there is an increased risk of the most critical adverse event associated with warfarin therapy, severe haemorrhage [1]. In a large study of adverse drug reactions (ADR) causing hospital admissions in Merseyside, England, warfarin was shown to be the third leading cause, responsible for just over 10% of all ADR-related hospital admissions [3].

Due to the difficulties in determining the eventual required stable maintenance dose (MD) for a given patient, many different regression models for MD prediction have been proposed worldwide. These models vary in terms of the predictive factors they include, with some including only patient demographics such as age, weight, height and co-morbidities. Other models include details of initial INR measurements, loading doses or drugs known to alter the effect of warfarin. Models comprising only demographic, loading dose or co-medication details use information that is readily available to the clinician and a few recent studies have derived such algorithms [4], [5]. More recently, dosing algorithms have also included genetic factors [6]–[10], specifically variants in the VKORC1 and CYP2C9 genes which have been shown to be associated with dosing requirements [11]. The benefit of including genetic information in dose prediction still remains unproven [12]–[14], although the science is conclusive that a patient's genetics alter their warfarin dose requirements[1], [11].

With a view to assessing how well previously published models predicted MD in a dataset outside the derivation dataset, we tested their predictive ability in two independent patient cohorts. This also allowed the performance of the models to be compared against each other. Further, it allowed us to evaluate how suitable the method of linear regression, the most commonly utilized method for deriving warfarin maintenance dosing algorithms, may be for patients at the maintenance dosing phase of warfarin therapy.

## Results

### Application of Selection Criteria

The selection process on the 21st of July 2014 found six dosing models, which meet the criteria specified in the Methods section of this manuscript, detailed in Table 1.

**Table 1:** List of Dose Prediction Models [4]–[6], [8], [15], [36].

Paper	Dosing Equation	1	2	3	4
Le Gal et al.	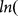 Dose 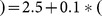 Total first week dose 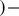 INR at day 8 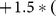 INR at day 5 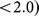	Y	Y	Y	Y
Solomon et al.	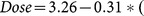 amiodarone 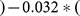 age 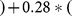 Loading Dose/end of load INR 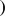	Y	Y	Y	Y
Anderson et al.	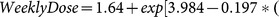 *1*2 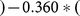 *1*3 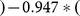 *2*3 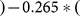 *2*2 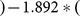 *3*3 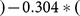 VKORC1 CT 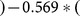 VKORC1 TT 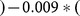 age 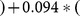 gender 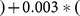 weight 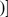 (25% dose reduction for those on amiodarone)	Y	Y	Y	U^a^
Wadelius et al.	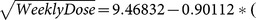 VKORC1 AG 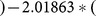 VKORC1 AA 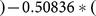 CYP2C9 *1*2 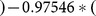 CYP2C9 *1*3 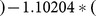 CYP2C9 *2*2 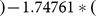 CYP2C9 *2*3 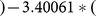 CYP2C9 *3*3 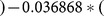 age 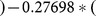 female 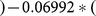 No. of drugs which increase INR 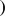	Y	Y	Y	Y
Sconce et al.	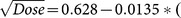 age 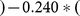 CYP*2 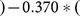 CYP*3 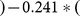 VKOR 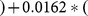 height 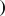	Y	Y	Y	Y
Zhu et al.	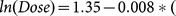 age 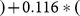 gender 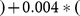 weight 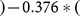 VKORC1-AA 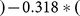 2C9-3 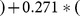 VKORC1-GG 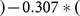 2C9-2 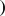	Y	N^b^	Y	Y

1. Was the paper published after 2003?

2. Does the model contain more than two di_erent variables than another dose prediction regression model already selected? (Although, where relevant novelty existed similar dose prediction regression models were compared and this novelty explained.)

3. Does the model include only covariates measured in the Liverpool study?

4. As R-squared is the most frequently reported statistic to judge model performance, is the value of this statistic above 0.5? Implying that more than 50% of the variability in the MD needs of the model's derivation cohort had been explained by the model.

a. R-squared statistic from derivation dataset not reported.

b. Reason for inclusion explained in manuscript.

The model presented by Le Gal et al. [5] includes only clinical covariates, including INR measurements on day 5 and day 8 and the total dose of warfarin taken during the first week. The second model, proposed by Solomon et al. [4] includes information on total loading dose, INR at the end of the loading phase, age and the use of the co-medication amiodarone, a well-known inhibitor of warfarin metabolism. These two models did not include any information on genotypes and, as a consequence, may have an advantage in that they are based on data readily available to the clinician, so can be used without having to attain a patients genotype information.

Four of the included models utilise genotypes for variants in CYP2C9 and VKORC1. The models proposed by Anderson et al. [7] and Wadelius et al. [15] assume that CYP2C9 alleles are non-proportional, thus including a separate covariate for each possible genotype, whereas the models proposed by Sconce and Zhu assume an additive effect of the variant allele. The models proposed by Anderson et al. [7] and Wadelius et al. [15] calculated a total weekly dose of warfarin; consequently clinicians would have to divide the recommended weekly dose into seven daily doses as they consider appropriate.

Anderson et al.'s model [7] also included demographic, genotype, and co-medication covariates. The model was applied in the randomized control trial and information on the models R-squared in the derivation cohort was not supplied. The model from Wadelius et al. [15], contained the largest number of covariates incorporating demographic and co-medication information alongside genotype covariates.

The model proposed by Sconce et al. [4] included less covariates than most of the other pharmacogenetic models, with demographic information only on age and height being included along with information on the genotypes. Similar in composition, but including weight instead of height, the model derived by Zhu et al. [6] has already been externally validated once in the recent study by Linder et al. [16], and was found to explain slightly less variability in the validation dataset than in the derivation dataset. The reason for the inclusion of two similar dosing equations was to assess whether the model developed by Sconce et al. [8] derived at the University of Newcastle, United Kingdom had an advantage over models derived in other countries in explaining variability in the Liverpool prospective validation cohort. The strength of these two models in particular was that they contain a small number of covariates yet explain a large amount of variability in their respective derivation datasets.

### Dose Prediction Model Performance

**Figure 1:**
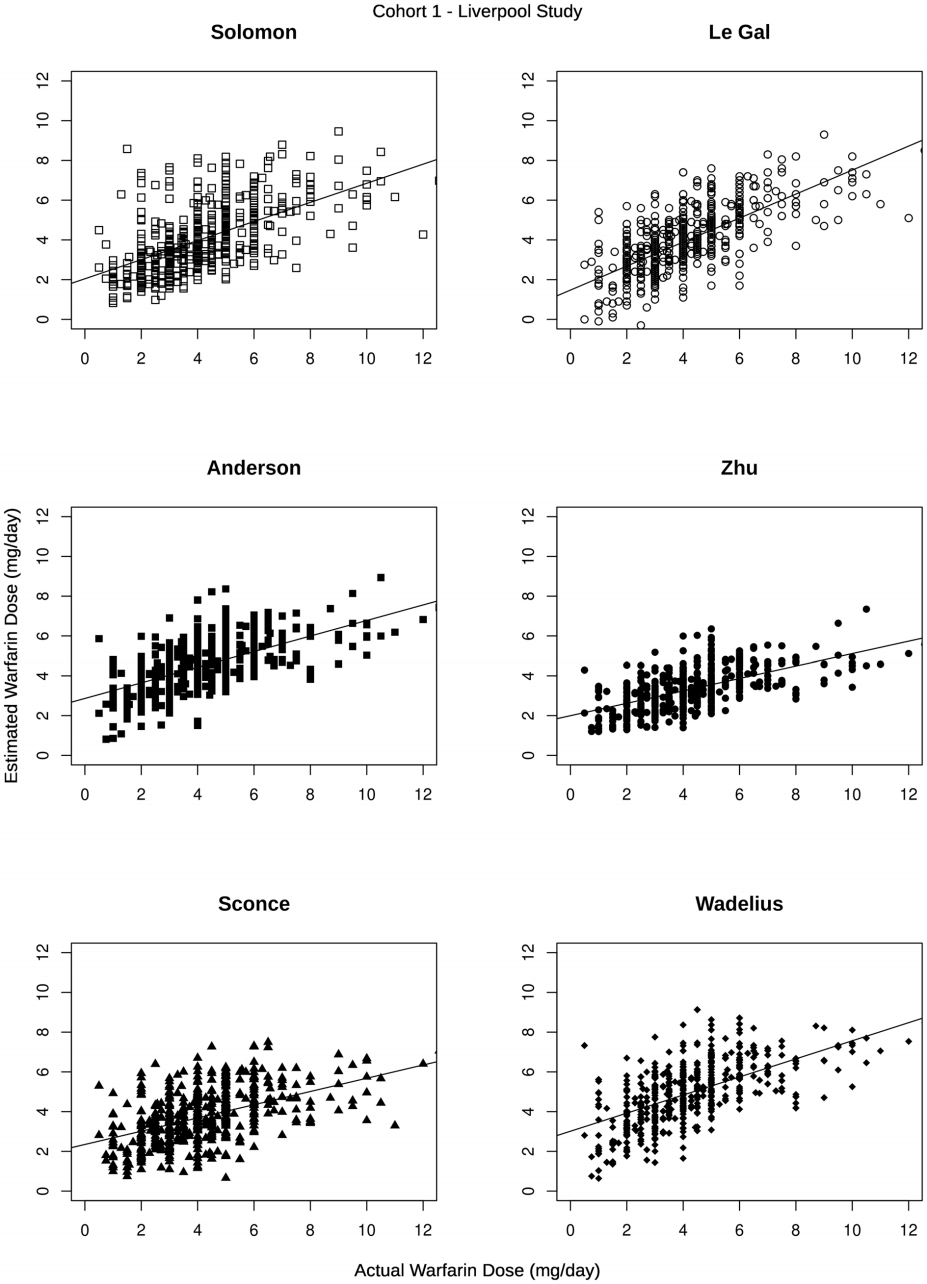
Graphs of predicted dose and actual warfarin dose in the Liverpool prospective study validation cohort.

**Figure 2:**
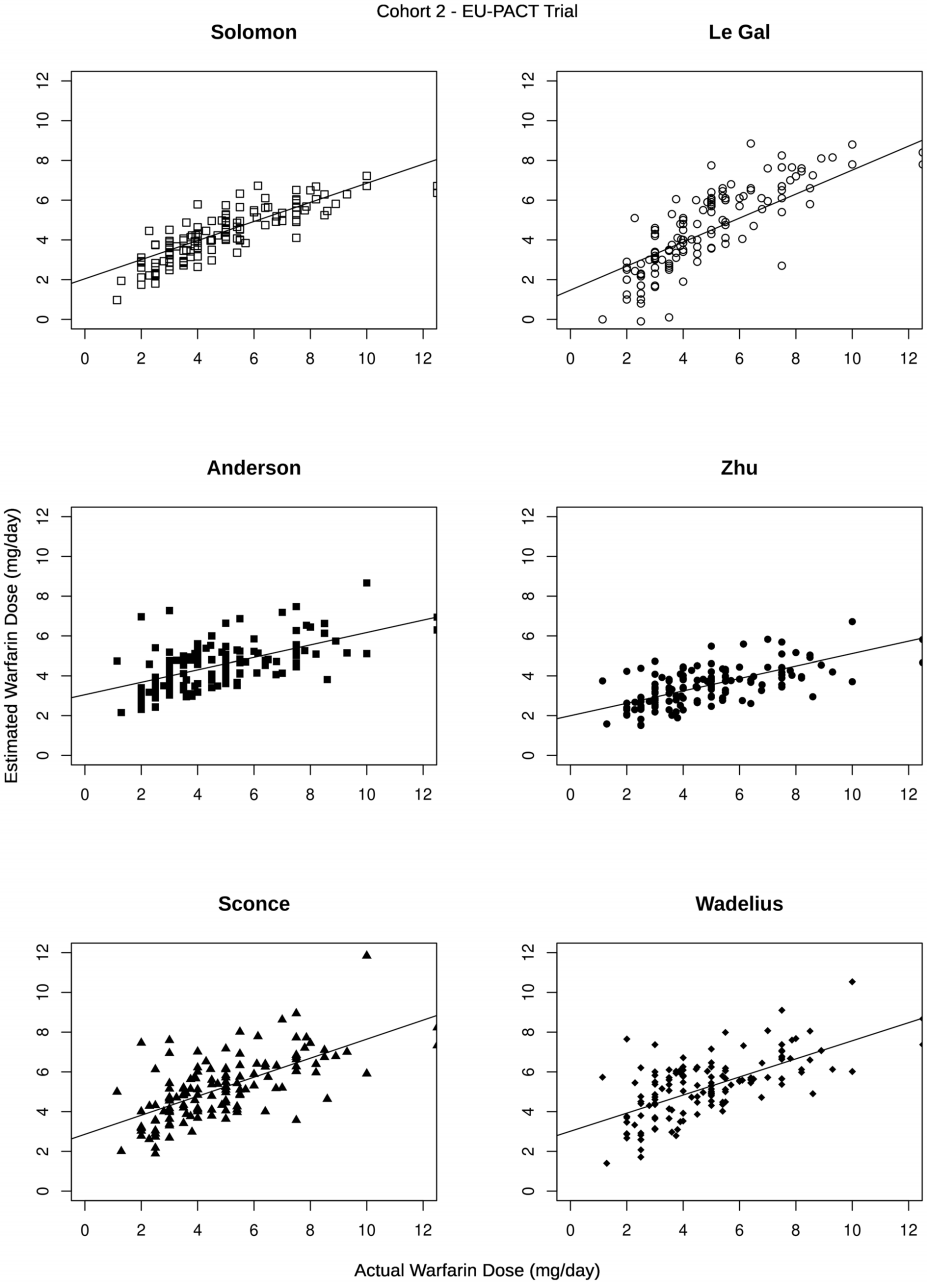
Graphs of predicted dose and actual warfarin dose in the EU-PACT trial control arm validation cohort.

The patient demographics, clinical information and pharmacogenetics in the LP and EUP validation cohorts is given in Table 2. The six selected models were applied to these two validation cohorts. Predicted versus actual stable MD for each validated model are shown in Figs. 1 and 2 for the Liverpool prospective (LP) and EU-PACT (EUP) validation cohorts respectively. Further summary statistics are presented in Table 3 and provide a deeper insight into the ability of the models to correctly estimate the required MD.

**Table 2:** Demographic, Clinical and Pharmacogenetic Information of Patient in the Validation Cohort.

Variable	Liverpool Study Patients				EU-PACT Trial Patients			
Number of Patients	508				133			
Age	Mean 68 years (SD 55–81)				Mean 67 years (SD 53–81)			
Gender	Male: 280 (55%); Female: 228 (45%)				79 (59%); 54 (41%)			
Weight (kg)	81.99 (SD 63–101)				86.00 (SD 64–108)			
Height (cm)	169 (SD 158–179)				170 (SD 160–180)			
Therapeutic Dose (mg)	4.19 (SD 2.14–6.24)				4.84 (SD 2.64–7.06)			
Amiodarone Co-medication	Yes: 46 (9%); No: 462 (91%)				Yes: 3 (2%); No: 130 (98%)			
CYP2C9		*1	*2	*3		*1	*2	*3
	*1	313	98	43	*1	84	26	12
	*2		4	9	*2		1	1
	*3			3	*3			0
VKORC1	GG	GA	AA		GG	GA	AA	
	206	224	77		59	48	16	

**Table 3:** Summary Statistics about the Performance of the Six Dosing Algorithms[4]–[6], [8], [15], [36].

**Cohort 1 - Liverpool Study**						
Model	Absolute Error		R-squared (%)		Intercept	Slope
	(Error)	(Percentage)	Coefficient of Determination	Adjusted		
	sMean ± SD	Mean ± SD				
Solomon	1.21±1.23	36.3±60.0	34.7	34.3	2.04	0.48
Le Gal	1.20±1.31	39.3±73.4	38.5	38.2	1.47	0.60
Anderson	1.18±1.13	39.7±68.5	38.6	37.3	2.86	0.39
Zhu	1.29±1.34	32.2±45.4	38.1	37.3	1.99	0.31
Sconce	1.32±1.31	37.4±60.1	24.9	24.2	2.35	0.33
Wadelius	1.37±1.19	46.7±83.2	36.2	36.2	3.01	0.46
**Cohort 2 - EU-PACT Control Arm**						
Model	Absolute Error		R-squared (%)		Intercept	Slope
	(Error)	(Percentage)	Coefficient of Determination	Adjusted		
	Mean ± SD	Mean ± SD				
Solomon	1.00±1.02	19.7±14.9	71.0	68.6	1.86	0.49
Le Gal	0.99±0.89	24.0±29.2	67.4	66.6	0.67	0.79
Anderson	1.38±1.16	32.3±37.8	34.5	29.1	3.05	0.31
Zhu	1.60±1.51	29.9±25.7	40.5	37.2	2.13	0.28
Sconce	1.28±1.10	33.4±43	43.8	41.6	2.86	0.48
Wadelius	1.33±1.11	36.3±48.8	42.1	40.7	3.12	0.45

The dosing model proposed by Le Gal et al. accounted for 39.2% (LP) and 66.6% (EUP) of the variability in stable warfarin MD along with a mean absolute error (MAE) of 1.20 mg/day (LP) and 0.99 mg/day (EUP), both around the desirable 1 mg/day [17]. The model predicted negative doses for some of the validation cohort. The reason why these patients were estimated as requiring a negative warfarin MD was a high INR or low dosages during the first week of treatment. The model does not have the ability to deal with these events and consequently the predicted doses for these patients are not applicable.

The model of Solomon et al. had a MAE statistic of 1.21 mg/day (LP) and 1.00 mg/day (EUP). However, the intercept terms of 2.04 (LP) and 1.86 (EUP) indicate overestimation occured at low doses.

The Anderson et al. model produced contrasting statistics in the two validation cohorts. The amount of variability explained was calculated to be 37.3%, the second highest in the LP validation cohort; yet in the EUP validation cohort this reduced to lowest amount explained of 29.1%. This contrast was also seen in the MAE and PAE statistics.

The Zhu et al. model produced estimates that had a mean absolute error of 1.29 mg/day (LP) and the amount of variability explained was calculated to be 38.1% (LP), the third highest of all models validated. However, the MAE of 1.6 mg/day indicates decreased performance in the EUP validation cohort

The Sconce et al. model explained the lowest amount of variability in the study with 24.2% in LP, though this improved to 41.6% in EUP, but all other statistics were on similar level in both validation cohorts.

The Wadelius et al. model prediction produced a PAE statistic of 46% (LP) and 36.3% (EUP) indicating that the model had large over or under predictions proportional to the actual dose. Further, the amount of variability explained was calculated to be 36.2% (LP) and 40.7% (EUP); the fourth highest of all models validated in both cohorts.

## Discussion

Several warfarin maintenance dosing algorithms have been published, many including pharmacogenetic information, in the form of linear regression models. Despite the number of published dose prediction regression models, they have rarely been integrated into standard clinical practice [12]. This in part, is due to the fact that most of these algorithms have not been externally validated in an independent dataset and if they have, then replication has been poor [16], [18], [19].

In this study, we have compared six different MD prediction linear regression models using two independent cohort of patients to test their predictive ability outside their original derivation cohorts. This was done by re-fitting each model in turn to two independent cohorts; a prospective patient dataset recruited in Liverpool, United Kingdom (LP) and the control arm from the EU-PACT trial (EUP).

Unsurprisingly, the performance of all six models was worse in the validation cohort as compared to the derivation cohort [16], [18]. The diminished performance could be explained by several factors. The two validation cohorts demographics shown in Table 2 reveal two points of note, firstly, the LP patients have a lower mean therapeutic dose than those in EU-PACT. This may be due to a difference in clinical practise between the two cohorts, potentially in the trade-off decision between maximising efficacy and minimsing adverse events. Secondly, the VKORC1 wild-type genotype is the most common in LP patients yet in EUP patients the homozygous dominant is more prevalent. Again, this difference could be due to different clinical practise provdided, particularly EUP patients without a VKORC1 variant allele reaching a stable maintenance dose with greater ease.

Examining the difference between our validation cohorts and the derivation cohorts for the six dosing algorithms investigated in this manuscript, there were differences that could contribute to differences in algorithm perfromance in validation. There is a range in the size of derivation cohorts ranging from Zhu et al.'s derivation cohort of 56 to Wadelius et al.'s derivation cohort of 850. The expectation would be for algorithms derived utilising smaller derivation cohorts to be able to explain less variability in a larger or more diverse cohort. This was not particularly evident in this study as Zhu et al.'s algorithm performed more strongly than Wadelius et al.'s algorithm in the larger LP validation cohort. In the EUP cohort the two algorithm's performances were relatively indistinguishable.

Differences in derivation cohort covariates can also contribute to altered algorithm performance in validation cohorts. For example, the Le Gal et al. (2010) and Solomon et al. (2004) derivation cohorts contain younger patients (mean ages of 58 and 55). Five out the of the six algorithms compared in this manuscript contain a coefficient for age. Curiously, the Le Gal et al. derivation cohort had a higher mean therapeutic dosage requirement to our validation cohorts, despite age being a factor that increases maintenance dose, shown in Table 1. This suggests that even though age has an effect on warfarin maintenance dose requirements, the relationship may not be linear in nature. This is reinforced by Moreau et al. (2011) [20] were elderly patients are shown to require lower induction and maintenance doses.

For warfarin dosing in peadiatrics, a number of specialised algorithms have been produced [21]–[24], further showing that age has a complex relationship with warfarin dosing requirements. In the more general population algorithms investigated in this manuscript, the age to dose relationship may not be as prevalent, however, further research is recommended to assess the linear relationship assumed by linear regression methods. Hamberg et al. (2014) [25] provide an excellent overview of current dosing algorithms available for paediatric populations, comparatively coefficients attributed to age are higher than those in this manuscript. Currently, paediatric algorithms use similar covariates to adult algorithms however future sources of variability found in either subpopulations are recommeneded to be investigated for translational impact.

The validation cohorts utlised in this manuscript consisted of various Caucasian populations. There have been a number of recent algorithms derived in non-Caucasian populations [26]–[28] and a study of the effect that the VKORC1 polymorphism across 3 racial groups [29] that show dose requirements vary with ethnicity.

The inconsistent replication observed in manuscript, which has also been observed previously [9], [18], leads us to hypothesise that regression modelling may not be the most optimal approach for developing a warfarin MD algorithm. Linear regression models may not be able to fully draw on the variability that can be explained by potential factors. Alternatively, potential factors may be important in different stages of warfarin therapy, but less consequential in the determination of a dose to prescribe a patient in the maintenance phase of therapy. This latter suggestion is hypothesised for pharmacogenetic factors in Horne et al. (2012) [30].

Different genetic biomarkers have been hypothesized to affect warfarin dose requirements; these include the genes, CYP4F2, CALU and GGCX [31]–[34]. The implementation of these new genetic biomarkers into dosing algorithms has been seen only in non-Caucasian derivation cohorts [26], [28], [31]–[33]. The validation cohorts utlised in this manuscript consisted of various Caucasian populations so we were unable to independently validate non-Caucasian algorithms in this manuscript. Further research of alternative genes hypothesised to affect warfarin dosing could be beneficial in Caucasian populations. This research should be in consideration of the current literature that shows the VKORC1 polymorphism causes differing warfarin dose requirements across 3 racial groups [29], potentially investigating epistasis effects.

Due consideration should be taken that, unlike randomized control trials, the method of validation performed in this paper does not allow for dose prediction models to be assessed by clinical endpoints. At the time of writing, there have been four randomised trials comparing different dose prediction models to standard therapy [7], [35]–[37]. The randomised control trial reported in Anderson et al. (2012) [36] compared two pharmacogenetic dosing algorithms to standard warfarin therapy; the results showed that pharmacogenetic-guided dosing was superior to standard warfarin therapy according to three clinical endpoints (percentage of out-of-range INRs, time in therapeutic range and adverse event rates). Pirmohamed et al. (2013) [37] concludes that pharmacogenetic-based dosing was associated with a higher percentage of time in the therapeutic INR range than was standard dosing during the initiation of warfarin therapy. Further, there were significantly fewer incidences of excessive anticoagulation in the genotype-guided group.

However, the results from the two other randomised control trials comparing dose prediction models to standard therapy, Anderson et al. (2007) [7] and Hillman et al. (2005) [35], did not conclude significant differences in any outcomes. This leads to an uncertainty about the true merit of algorithms when compared with standard care. Importantly, whilst further trials comparing an algorithm guided dosing arm to standard therapy are recommeneded, the comparison is complicated by the subjective interpretation of standard therapy. With increasing numbers of RCTs being conducted for comparing these specific arms there may be the opportunity to meta-analyse the results to conclude the between study variability and, potentially, the causes of this variability.

The randomised control trial, Clarification of Optimal Anitcoagulation through Genetics (COAG), had an alternative set of arms, patients dosed according to combined clinical and pharmacogenetic algorithms and patients dosed according to clinical only algorithms. The results showed that there was no significant difference between the two patient arms for multiple outcomes. The outcomes measured in COAG were time in therapeutic range, any INR readings above 4, major bleeding or thromboembolism, time to first therapeutic INR, the time to the determination of a maintenance dose and the time to an adverse event. The conclusions from this study require a different prespective on ascertaining the merit of warfarin maintenance dosing algorithms; as there were no significant differences observed between endpoints observed in the respective pharmacogenetic and non-pharmacogenetic dosing algorithm arms, a critical view of the algorithms themselves is possible. Two potential conclusions can be proposed, the extra sources of variability included in the pharmacogenetic dosing algorithm do not aid dosing in comparison to the non-pharmacogenetic algorithm or the algorithm methodology, linear regression, does not maximally implement the extra sources of variability. These two conclusions are also proposed by this manuscript, where, similarly, pharmacogenetic algorithms outcomes are relatively indistinguishable from non-pharmacogenetic algorithms.

A further large, well powered trial, Genetics Informatics Trial (GIFT) is currently underway to investigate clinical endpoints and respective clinical [38]; this will provide further insight into targeted warfarin maintenance dosing.

**Figure 3:**
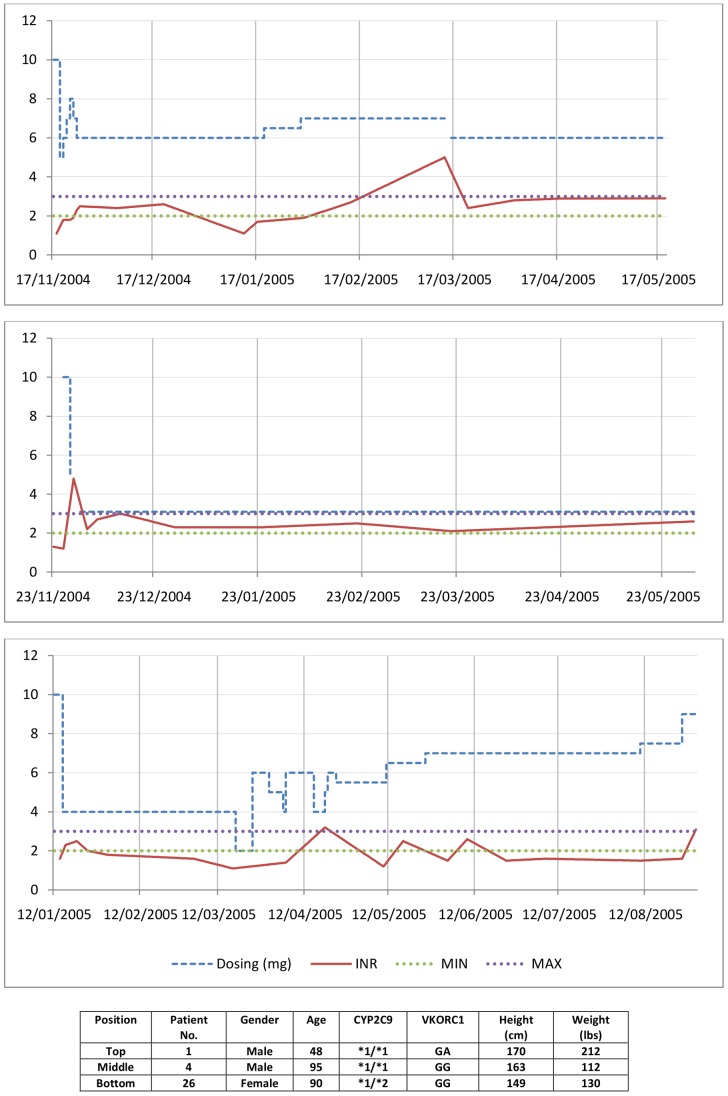
INR-Time profiles of three patients receiving standard care.

The limitations of linear regression methods should be considered when concluding which methodology to utilise for deriving MD prediction. When developing MD prediction regression models, the outcome of interest is stable MD; therefore it is necessary to identify, for each patient included in the dataset used to derive the model (the derivation dataset), the dose at which stable, in-range INR has been achieved. However, since INR is sensitive to many factors, including dietary changes [39] and alcohol intake [9], measurements often fluctuate out of range even after an initial period of stability has been achieved. Fig. 3 shows three different patients from the LP cohort all receiving standard care and, in all but one patient, INR measurements do not continuously stay within therapeutic range.

Developing a regression model necessitates each patients stable MD to be determined in accordance with a particular definition of stable dose. Choosing this definition in itself is difficult as evidenced by the many different definitions given in the literature (see Table 4 in Jorgensen et al. (2012) [40] and Section S2, Supplementary Appendix 1 in Klein et al. (2009) [41]), and stable dose of a patient under one definition may well be different to the patients stable dose under another. The derivation cohorts of the six dosing algorithms studied in this manuscript utilised a similar three visit based definition of steady dose. The exceptions were Le Gal et al. [5] and Anderson et al. [7]. Le Gal et al. defines warfarin therapy as the dose that kept INR measurements in range during days 18–28 after therapy intiation. Anderson et al. original derivation cohort stable dose defintion, found in Carlquist et al. (2006) [42], required the patient to be within therapeutic INR range for one month.

Depending on the definition used, patients are excluded from analysis on the basis that their dosing history never meets the criteria specified in the definition. In the validation cohorts of this study, stable dose was defined as three consecutive INR measurements within the individual's target range, at the same daily dose. Due to frequent fluctuations in and out of range, and corresponding dose changes, the dosing history of 575 patients did not meet this criterion and therefore had to be excluded from analysis. Not only is this a significant loss of information, but more importantly it might lead to important sources of variability being missed since the least stable patients are excluded from analysis. Therefore, dose prediction regression models can overlook information needed to appropriately recommend doses for the least stable patients - exactly the patients who need individualized dosing. Further, when different stable dose defintions are utilised in derivation cohorts, the coefficients found significant and their values may be altered due to differences in the subsequent data. This means that performance in validation cohorts with different stable dose defintions can be affected. Potentially this has been observed in this study with the Anderson et al. algorithm performing inconsistently between validation cohorts. On the other hand, the Le Gal et al. algorithm shows less signs of reduced performance.

Methods such as those implemented in Hamberg et al. (2010) [43] and Perlstein et al. (2011) [44] incorporate non-stable patients into the analysis as well as looking at pharmacokinetic (PK) and pharmacodynamic (PD) parameters. Further research has been undertaken to explain the variability in PK/PD parameters for warfarin in various papers [8], [16], [43], [45]–[47]. PK/PD modelling along with the study of the variability in PK/PD parameters endeavour to explain the non-linear relationship between warfarin dose and response [43], [46]. Deriving information on patients PK/PD parameters allows for more adaptive models which in turn may improve the individualisation of warfarin therapy for patients. Adaptive models would ideally provide initial dose recommendations, based on demographics, genotype and other readily attainable information, not too dissimilar from regression algorithms, and response could then be fed back into the model regularly, with the model eventually providing updated MD predictions.

In summary, validation of dosing algorithms in an independent dataset is paramount for any warfarin dose prediction regression model and, as we have demonstrated, the predictive ability of a model diminishes in an independent dataset as compared to a derivation dataset. Further, the performance of an algorithm from one cohort to the next is uncertain, as shown by the comparison of the results in the LP and EUP validation cohorts. Notably the pharmacogenetic MD regression models showed less consistency in performance than the non-pharmacogenetic algorithms.

Further, we have demonstrated that a significant proportion of patients are excluded from the datasets used to derive linear regression dose prediction models since the criteria set out in the definition of stable dose precludes them. This can have a detrimental effect on model performance since it means that information needed to appropriately recommend doses for the least stable patients may be overlooked. In light of these issues, we suggest that more advanced methods of developing dosing algorithms should be explored. In particular, these methods should not assume a single value of stable dose for a patient, but rather allow dose requirements to adapt with time, to reflect the sensitivity of INR to variation such as dietary changes, alcohol intake and co-medications. Validation in multiple cohorts is very much recommended to ascertain the consistency of a warfarin maintenance dosing algorithm

## Methods and Materials

### Ethics Statement

Approval was obtained from the Birmingham South Research Ethics Committee to recruit patients for a previous study (“the Liverpool prospective study” [48]). Patients consented in writing to participate in the study, which included both the provision of samples (DNA, serum and plasma), and their analysis, that could then be linked to their clinical data through a study identification (ID) tag. Using the Study ID tag, assigned to the patient prior to the collection of clinical data and samples, maintained anonymity of patient data from the authors of this paper. The analysis provided at the beginning of the Liverpool study covered all the analyses performed in this paper. The collective authors can confirm that there were no additional data collected for the current study, the Liverpool study data was sufficient for our analysis.

The EU-PACT warfarin trial was a pragmatic, single-blind, randomized, controlled trial that was designed to determine whether genotype-guided warfarin dosing was superior to standard dosing. The trial methods have been described previously [49]. The protocol (available with the full text of the Pirmohamed et al. (2013) article [37] at NEJM.org) was approved by the local research ethics committee in Liverpool, United Kingdom, and by the regional ethical review board in Uppsala, Sweden. Oversight was provided by a data and safety monitoring board. Data were collected by the investigators and were analyzed by a statistician, who vouches for the accuracy and completeness of the data reported. All the Pirmohamed et al. (2013) [37] authors vouch for adherence of the study to the protocol. LGC (formerly the Laboratory of the Government Chemist) provided the point-of-care genotyping assay with funding from the European Union. The collective authors can confirm that there were no additional data collected for the current study, the EU-PACT study data was sufficient for our analysis.

### Validation Cohort

Patients were recruited from the Royal Liverpool and Broadgreen University Hospitals Trust and University Hospital Aintree between November 2004 and March 2006. Patients were required to be initiating warfarin therapy. Since the study was of an observational nature, patients received customary clinical care and dosing was in line with standard protocol within the recruiting hospitals. At the patients index visit, demographics and baseline INR were recorded, and a blood sample was taken for genotyping. Three further fixed study visits were scheduled for 1, 8 and 26 weeks after initiation onto warfarin. All INR values measured and dose changes made during the follow-up period were recorded. Three patients fitted with mechanical prosthetic heart values, and therefore had a recommended INR range of 3–4, were excluded from the current study, leaving a total of 997 patients from the initial one thousand. Full information on the procedure for the genotyping of patients is presented in the original paper on the study [48].

For the EU-PACT trial [37] patients were recruited in the United Kingdom (three centers) and Sweden (two centers). Eligible patients had not received previous treatment and required anticoagulation with warfarin with a target INR of 2.0 to 3.0. All participants gave written informed consent before taking part in the EU-PACT trial. Patients were randomly assigned to either the genotype-guided dosing group or the standard dosing (control) group. For the purpose of this validation paper, only the control arm of 216 patients was included to avoid bias of patients in the genotype-guided dosing group. In the control arm, patients 75 years of age or younger received 10 mg of warfarin on day 1, 5 mg on day 2, and 5 mg on day 3, whereas patients older than 75 years of age received 5 mg per day on days 1 through 3. The doses on days 4 and 5 and thereafter were determined according to usual local clinical practice. All patients were followed for 3 months, with INR measured on days 1, 4, 6, 8, 15, 22, 57, and 85. Some patients had additional clinic visits and INR measurements, but these were determined by clinical need.

In this validation study, stable MD was defined as the mean daily dose required to achieve three consecutive INR measurement within the individuals target range (2–3 for patients that formed our validation cohort), at the same daily dose. Stable MD was identified on the assumption that patients fully complied with their dosing regimens and that the only reason for dose modification was recorded clinical practice. After applying this definition of stable MD, 508 patients from the Liverpool prospective study (LP validation cohort) and 133 patients from EU-PACT control arm (EUP validation cohort) were found to have achieved stability during follow-up and they formed the subset of patients included in the validation cohort. A summary of patient demographics, clinical information and pharmacogenetics in the LP and EUP validation cohorts is given in Table 2.

### Choosing the models to validate

The intention was to obtain a set of dose prediction regression models which were relatively recent, sufficiently diverse from each other, applicable to our validation cohort and had high performance in their original derivation dataset. Models were not considered from publications over ten years old which meant that the majority incorporated pharmacogenetic information [11]. Variables included in the models included a unique combination of any of the following types demographic, clinical, pharmacogenetic, and dose response and co-morbidity information. Applicability to the validation cohort was mandatory; this meant models selected would need to consist of covariates measured in the Liverpool prospective study and the EU-PACT trial. The models initial performance in the derivation dataset was also highly important, on the basis that algorithms tend to predict less of the variability in validation datasets [16], [18]. The Liverpool prospective trial patient data were included in the IWPC derivation cohort therefore, the two algorithms presented in Klein et al. (2009) [41] were excluded from the search to remove any bias that could occur from their inclusion.

### Statistical analysis

To determine the ability of the six regression models to explain variability in MD requirements, the warfarin MD predicted by the regression model was plotted against the actual warfarin MD in the two validation cohorts and then a linear regression line fitted. The accuracy of the algorithms was judged using the R-squared statistic (unadjusted and adjusted), mean absolute error (MAE), mean percentage absolute error, and the slope and intercept of the regression line.

The R-squared statistic is a measure of the amount of variability explained in a dataset. However, the R-squared statistic is not affected by a constant error in dose prediction; for example, if we altered any algorithm in this study by adding 100 mg to each dose prediction, the R-squared statistic would remain the same even though the dose predictions would now be severely over estimated. The mean absolute error (MAE) statistic measures how close the predictions are to the actual values across all patients in a dataset, and therefore is important when considering which model has the best predictive capability. However, the clinically desired value of this statistic varies. For example, Kimmel [17] recommended a MAE of 1 mg/day as a change in warfarin dose from a baseline of 5 mg is sufficient to change the INR by 0.5. The slope and intercept of the R-squared line are also measures of a models accuracy, a slope of one and an intercept of zero indicate that there is no proportional error and no constant error respectively. If the slope coefficient is different from one there will be either over or under prediction in some, if not all estimated doses. The intercept term gives an insight into how well the model is predicting at low doses. These statistics should all be used together to appropriately judge a model as each statistic can appropriately assess a different aspect of the models predictive ability.
